# A Unified Framework for Survival Prediction: Combining Machine Learning Feature Selection with Traditional Survival Analysis in Heart Failure and METABRIC Breast Cancer

**DOI:** 10.3390/diagnostics16050790

**Published:** 2026-03-06

**Authors:** Fangya Tan, Jian-Guo Zhou, Shuqiao Li, Bowen Long, Srikar Bellur, Yang Zhou, Mark Newman

**Affiliations:** 1 Department of Analytics, Harrisburg University of Science and Technology, Harrisburg, PA 17101, USA; ftan1@my.harrisburgu.edu (F.T.); blong@alumni.harrisburgu.edu (B.L.); sbellur@harrisburgu.edu (S.B.); mark@trinetteandmark.com (M.N.); 2 Department of Oncology, The Second Affiliated Hospital of Zunyi Medical University, Zunyi 563000, China; jianguo.zhou@zmu.edu.cn; 3 Department of Physics, The Graduate Center, City University of New York, New York, NY 10016, USA; yzhou4@gradcenter.cuny.edu

**Keywords:** survival analysis, machine learning, feature selection, Gradient Boost Machine, Random Survival Forest, heart failure, METABRIC breast cancer, hazard ratio, Kaplan–Meier Curve, clinical trials, Cox model

## Abstract

**Background**: The clinical use of machine learning (ML) in survival analysis is often limited by the “black box” nature of complex algorithms, which makes their results difficult to interpret in practice. In this study, we propose a unified and clinically grounded framework that integrates ML-based feature selection with traditional survival analysis. This approach aims to bridge the gap between strong predictive performance and clear, clinically meaningful interpretation. **Methods**: High-impact prognostic clinical features were identified using ML models GBM-Cox, RSF, and LASSO-Cox with 5-fold stratified cross-validation and subsequently validated using Cox Proportional Hazards and Kaplan–Meier analysis. The framework was evaluated across two distinct disease domains, Heart Failure and the METABRIC breast cancer cohort, to assess robustness and generalizability. **Results**: In the Heart Failure dataset, age group, serum creatinine, and blood pressure stratified patients into distinct risk groups. The high-risk group had significantly higher mortality (HR: 2.61; 95% CI: 1.42–4.78; *p* = 0.0013). In the METABRIC cohort, age at diagnosis, HER2 status, and Nottingham Prognostic Index (NPI) showed strong survival separation (*p* < 0.001). The high-risk group had an HR of 2.73 (95% CI: 2.34–3.19) and the faced a significantly shorter median survival (104.7 vs. 252.3 months), representing a 12.3-year reduction in life expectancy compared to low-risk group. This prognostic separation emphasizes the predictive power of selected baseline variables. Performance remained stable across cohorts, with C-index values (0.665–0.794) consistent with standard clinical benchmarks. **Conclusions**: Integrating cross-validated machine learning feature selection with Cox-based survival analysis enables stable and clinically interpretable risk stratification across diseases. By translating ML selected predictors into hazard ratios and absolute survival differences, this framework provides a reproducible and clinically grounded approach for survival risk assessment.

## 1. Introduction

Survival analysis is a foundational tool in clinical research, used to model time-to-event outcomes such as disease progression, treatment response, and patient mortality [1,2]. Traditionally, it has been most widely applied in oncology [3,4,5], where time-to-death metrics are critical for evaluating treatment efficacy. More recently, survival analysis has expanded into other therapeutic areas, including cardiovascular disease [6,7,8], liver cirrhosis [9,10,11], and chronic conditions where long-term outcomes or organ failure risks are key concerns. Common methods such as the hazard ratio (HR) and Kaplan–Meier (KM) curves are central to these analyses, enabling clear comparisons between patient subgroups and supporting both regulatory decisions and patient treatment strategies based on risk [2,12,13].

In parallel with classic statistical approaches like HR and KM, the rise of electronic health records and high-dimensional data has driven rapid adoption of machine learning (ML) in healthcare. ML techniques, ranging from embedding Cox proportional hazards extensions [14] to Random Survival Forests (RSF) [15], Gradient Boosting Machines (GBM) [16], XGBoost [17], and CatBoost [18], have been increasingly used to monitor chronic diseases, identify early biomarkers [19], and predict clinical outcomes such as adverse events [20] and overall survival [21]. These models excel at handling high-dimensional and longitudinal data, making them well-suited for complex prediction tasks. However, their “black-box” nature often limits clinical interpretability, particularly when trying to link model-identified features with actionable, patient-level disease guidance.

By contrast, traditional statistical methods such as the hazard ratio offer clear, interpretable risk comparisons between groups (e.g., treatment vs. placebo) at any time point [5]. Kaplan–Meier curves further illustrate survival probabilities over time and allow for intuitive visualization of group differences [22]. For example, a study of 3878 breast cancer patients treated in Edinburgh over a 20-year period used HRs and KM curves to show that median survival declined from 10 years (Stage I) to less than 1 year (Stage IV), with mortality differences by stage diminishing after 5 years. Another study demonstrated improved survival following breast cancer recurrence from 1974 to 2000 and identified Estrogen Receptor (ER) status as a key prognostic factor, such that ER-negative patients had a 71% higher risk of death (HR = 1.71) than ER-positive patients (*p* < 0.001) [23].

Similarly, in non-small cell lung cancer, HRs showed that low serum albumin (<35 g/L) was associated with a 42% reduction in risk (HR = 0.588), while ≥10% weight loss increased mortality risk by 62% (HR = 1.62) [24]. These examples highlight the enduring value of traditional survival analysis: it provides quantifiable, interpretable risk estimates supported by visual summaries like HR and KM curves.

This principle of using interpretable metrics is foundational even in large-scale evidence synthesis. For example, the systematic review by a comprehensive study [9], analyzing 118 studies, used aggregated evidence from HR-based models to establish key prognostic factors in cirrhosis, such as serum creatinine, total bilirubin, and INR. A key question is whether machine learning models trained on raw clinical data would also identify these well-established predictors or instead focus on less interpretable features.

As the volume of available clinical variables grows, including labs, vitals, and comorbidities, it becomes increasingly labor-intensive to manually test each predictor using traditional regression. Biostatisticians have turned to variable selection techniques such as LASSO [25] to address this issue. Yet, LASSO has notable limitations in capturing the complex structure in high-dimensional clinical data. Because its penalty treats predictors independently and does not explicitly account for correlation, LASSO tends to arbitrarily select one variable from groups of correlated features while excluding others. This behavior can lead to unstable feature selection, reduced reproducibility, and the omission of clinically meaningful predictors that convey complementary information [26].

In recent years, ML has gained prominence in clinical research due to its ability to handle high-dimensional data and uncover complex patterns. Its computational power has encouraged researchers to apply it in identifying prognostic features that could guide treatment decisions. For example, study [20] demonstrated that biomarker categories could predict immune-related adverse events (irAEs), offering a way to stratify patients who are more likely to benefit from immunotherapy in non-small cell lung cancer. However, while this study effectively identified predictive biomarkers, it did not explore how irAEs or those biomarkers relate to overall survival outcomes.

Similarly, the EURAMOS-1 [21] study evaluated a rare cancer (osteosarcoma) using eight clinical variables to compare traditional Cox models with Random Survival Forests (RSF) and Survival Neural Networks (SNN), focusing on predictive performance via the concordance index (C-index). In this relatively low-dimensional setting (~2000 patients, 8 variables), Cox models performed comparably to more complex machine learning methods. However, the study did not report hazard ratios or offered insights into the clinical significance of the selected features.

These studies collectively highlight a key limitation in many ML applications in medicine: uncertainty around the clinical interpretability of the features these models identify. While ML tools such as SHAP values can rank variables by their predictive influence, it is often unclear whether these features also exhibit statistically consistent and interpretable effects, such as those reflected by HR, and the KM curve in traditional survival analysis. Therefore, the degree to which ML-derived predictors align with established clinical risk factors remains a critical but unresolved question.

Some studies have attempted to bridge this gap. For example, in cardiovascular disease research, machine learning models have successfully highlighted serum creatinine and ejection fraction as key predictors of survival outcomes [6]. Although these variables are clinically meaningful, the analyses are typically limited to a single disease area, raising concerns about their applicability across broader, more diverse therapeutic areas. Other research [27] integrated ML with conventional methods, such as logistic regression, Cox models, Random Forest, support vector machines (SVM), and neural networks (NN), to explore risk stratification. Yet these studies often stopped short of examining survival time explicitly or providing interpretable outputs, which limits the usefulness of such methods for clinical decision-making.

Further work [28] expanded the input space to include coexisting medical conditions like hypertension and diabetes, alongside laboratory variables. While this approach improved predictive performance, raising the AUC by 11% across Cox and RSF, it primarily focused on model accuracy on overall survival event rate rather than detailing the clinical relevance of individual predictors. Even large-scale studies, such as one involving over 468,000 patients [25], which compared models including RF, GBM, XGBoost, CatBoost, SVM, LASSO, and extreme learning machines (ELM), emphasized overall survival model prediction performance. Although this study correctly identified ejection fraction, serum creatinine, and blood urea nitrogen (BUN) as top features and effectively stratified patients by risk, it did not present KM plots or HRs to support the clinical impact of those variables.

The gap in machine learning models highlights predictive features but often lacks clinical evidence to explain their impact, as is also evident in breast cancer research using the METABRIC dataset. For example, study [29] applied Cox proportional hazards (Cox PH), RSF, and conditional inference forests (Cforest), the models consistently identified age, tumor size, and number of positive lymph nodes as important prognostic features. Although these variables appeared influential in the models, the study did not provide supporting evidence, such as HR, to quantify their survival impact.

Several recent studies have explored the application of machine learning (ML) methods to breast cancer survival prediction, often comparing them with traditional Cox proportional hazards (PH) models. Studies [30,31] employed Cox PH, RSF, and DeepHit to evaluate survival outcomes. Classical factors such as estrogen/progesterone receptor (ER/PR) status, tumor stage, and age remained significant in Cox regression, with hormone receptor positivity associated with lower mortality risk. While the study reported *p*-values for these features, explicit hazard ratios (HRs) were not provided, limiting assessment of effect magnitude. As a result, while findings aligned with clinical expectations, quantitative validation through traditional survival metrics was incomplete.

The study [32] took a more model-diverse approach, using GBM, RSF, SVM, and ANN to predict five-year survival, reporting an overall AUC of 0.67. Clinically relevant variables such as the Nottingham Prognostic Index (NPI), tumor size, stage, ER/PR/HER2 status, and surgery type were emphasized. Similarly, study [33] showed that explainable ML models (XGBoost with SHAP values) outperformed Cox regression in C-index performance, identifying tumor stage, age, hormone receptor status, HER2, and treatment as key predictors. Despite improved discrimination and interpretability, neither study linked these predictors to conventional outputs such as Kaplan–Meier (KM) curves or HRs, limiting clinical interpretability beyond model accuracy.

A more integrative analysis was presented in [34], which compared Cox PH, RSF, gradient-boosted survival (GBS), and survival support vector machines (SSVM). In this study, ML models, particularly RSF, outperformed Cox PH in predictive performance, especially when combining clinical and transcriptomic features. SHAP analysis highlighted both established clinical factors (e.g., age) and novel genomic markers (e.g., ERAS, SLC14A1, LCN15). However, hazard ratios and survival curves were not reported, constraining validation within a clinically interpretable survival framework.

Across these studies, a consistent limitation emerges: although machine learning (ML) models effectively identify predictive features and achieve strong accuracy in survival prediction, they often lack grounding in traditional survival metrics. The absence of hazard ratios (HRs) and Kaplan–Meier (KM) curves makes it difficult to determine whether model-selected features are statistically robust, clinically meaningful, and actionable in practice. As a result, many ML studies improve predictive performance within a single disease domain but fail to validate findings using clinically trusted measures or demonstrate broader translational value. This disconnect remains a key barrier to clinical adoption.

To bridge this gap, we propose a unified framework that integrates ML-based feature selection with traditional survival analysis. In this framework, ML is used to identify high-impact predictors, and these features are subsequently validated using established survival metrics, specifically hazard ratios and Kaplan–Meier curves. This integration provides two major gains. First, it enables time-quantified clinical benefit by translating model outputs into interpretable survival differences (e.g., survival probability over time). Second, it allows direct numerical comparison of effect sizes through hazard ratios, ensuring that ML-selected features can be evaluated using familiar statistical standards. Together, this approach strengthens both interpretability and clinical trust.

To demonstrate generalizability, we apply this framework across two chronic diseases: heart failure and breast cancer. These conditions were selected due to their high global burden, biological heterogeneity, and the importance of early risk stratification. All datasets were chosen for quality, transparency, and public availability to ensure reproducibility.

Heart failure remains a leading cause of cardiovascular mortality worldwide, accounting for an estimated 17.9 million deaths annually [35]. Early identification of high-risk patients is essential to guide therapy and reduce hospital readmissions. We use a dataset of 299 patients collected from the Faisalabad Institute of Cardiology and Allied Hospital in Pakistan between April and December 2015 [36].

Breast cancer is the most frequently diagnosed cancer among women and the second leading cause of cancer-related mortality globally [37]. Despite advances in treatment, substantial survival disparities persist. The METABRIC dataset provides comprehensive molecular and clinical data for approximately 2509 patients, enabling integrated genomic and survival analysis [38].

By validating ML-identified predictors using hazard ratios and Kaplan–Meier curves, this study establishes a clinically grounded and statistically transparent framework. Rather than replacing traditional methods, the approach aligns ML with established survival analysis standards, facilitating translation into real-world clinical workflows.

## 2. Materials and Methods

### 2.1. Study Dataset, Population, and Data Preprocessing

This study utilized two publicly available datasets: a Heart Failure clinical records cohort comprising 299 patients with 11 features [36] (Table 1) and the METABRIC breast cancer cohort, including 1310 patients with 20 features after data cleaning (Table 2) [38]. The Heart Failure dataset contains complete clinical records without missing values. To ensure methodological consistency and data integrity across both datasets, a complete case analysis (CCA) approach was applied to the METABRIC cohort. Records with missing values were excluded so that machine learning benchmarking was performed exclusively on observed clinical data, thereby avoiding potential bias or uncertainty introduced by imputation procedures.

The primary outcome for both datasets was overall survival (OS), defined as time from diagnosis to death from any cause or censoring at last follow-up. As METABRIC contains multiple time-to-event endpoints, relapse-related outcomes were excluded to maintain endpoint consistency and avoid overlapping survival definitions.

Non-informative identifiers, such as subject ID and cohort indicators, were removed prior to analysis. Variables with no variability or limited contribution to model performance were also excluded; specifically, cancer type and sex were removed from the METABRIC cohort due to their uniform distribution.

### 2.2. Survival Prediction Modeling

Survival modeling methods specifically designed for right-censored time-to-event data were employed, with outcomes defined by survival time and a binary event indicator (death vs. censoring). Model-derived risk groups were subsequently evaluated using Kaplan–Meier estimation and log-rank testing (via the survival and survminer packages) to provide clinical validation of risk stratification.

To ensure robust evaluation, each dataset was partitioned into an 80% training set and a 20% independent test set using stratified sampling to preserve the proportion of events and censored observations. Model development was conducted within the training set using 5-fold stratified cross-validation for hyperparameter tuning and feature selection. In each iteration, four folds were used for model fitting and one-fold for validation, with event proportions preserved to reduce overfitting. Final model performance was evaluated on the held-out test set to provide an unbiased estimate of generalizability. No artificial resampling or synthetic balancing of clinical covariates was applied; the natural distribution of patient characteristics was preserved to reflect the underlying clinical population.

Three machine learning approaches with established utility in survival analysis were evaluated using R statistical software (version 2025.09.2): Gradient Boosting Machine with a Cox loss function (GBM-Cox) [39], Random Survival Forest (RSF) [15], and LASSO-regularized Cox regression [40]. Tree-based ensemble models, including GBM and RSF were selected for their ability to model non-linear effects and complex interactions, whereas LASSO-Cox provided a regularized extension of the traditional Cox model, enabling simultaneous feature selection via coefficient shrinkage.

The GBM-Cox model was implemented using the gbm package with 1000 trees, interaction depth of 3, and a learning rate of 0.01 to promote stable convergence. The RSF model was fitted using the randomForestSRC package with 1000 trees and the log-rank splitting rule, and variable importance was assessed using permutation measures. LASSO-Cox models were implemented via the glmnet package, with the optimal regularization parameter selected through internal cross-validation, family = cox, and alpha = 1; variable importance was defined by the absolute magnitude of regression coefficients.

Deep learning-based survival models (e.g., DeepSurv, DeepHit) [41,42] were not included in this analysis. Given the modest sample sizes and structured clinical variables, tree-based and regularized Cox models were considered sufficient for stable estimation and interpretability.

### 2.3. Model Performance Evaluation

Model performance was assessed using three complementary survival metrics: Harrell’s concordance index (C-index) [43], time-dependent area under the curve (AUC) [43], and the Integrated Brier Score (IBS) [44]. Harrell’s C-index was calculated as an overall measure of discrimination across the full follow-up period. Time-dependent AUC was used to assess discrimination at clinically meaningful horizons. In the Heart Failure dataset, AUC was evaluated across all follow up period to ensure stable estimation. In METABRIC, AUC was assessed at the 60-month (5-year) horizon, consistent with oncological benchmarks.

The Integrated Brier Score (IBS) was used to quantify overall prediction error and calibration. IBS was evaluated across 75% event occurrence in Heart Failure and at 60 months in METABRIC to ensure stability under right-censoring. All performance metrics were averaged across 5-fold cross-validation, and 95% confidence intervals were derived from fold-wise variability using the t-distribution.

Since the primary objective of this study was robust and clinically interpretable feature identification rather than maximal predictive optimization, hyperparameter tuning was therefore performed to achieve reasonable discrimination without pursuing exhaustive performance maximization. Importantly, key predictors remained stable across models with varying discrimination levels, indicating that feature selection was not driven by marginal gains in predictive metrics.

### 2.4. Feature Importance and Clinical Translation

To translate machine learning outputs into interpretable clinical insights, feature importance scores were extracted from the GBM, RSF, and LASSO models. Variables that were consistently highly ranked across models or strongly prioritized within a specific disease context were selected as candidate predictors for downstream clinical validation.

To evaluate these predictors within a conventional survival analysis framework, continuous variables were categorized into clinically meaningful subgroups according to established medical criteria. This categorization enabled estimation of hazard ratios (HRs) and formal assessment of the proportional hazards (PH) assumption using Schoenfeld residual tests. Categorization criteria were defined as follows:

Heart Failure Dataset:

- Age at diagnosis: <65 vs. ≥65 years [45].
- Left Ventricular Ejection Fraction (LVEF): Normal (41–75%) vs. Abnormal (<41% or >75%) [46].
- Serum Creatinine: Normal (0.59–1.04 mg/dL for females; 0.74–1.35 mg/dL for males) vs. Abnormal [47].

METABRIC Breast Cancer Dataset:

- Age at diagnosis: <65 vs. ≥65 years [45].
- Nottingham Prognostic Index (NPI): Good (≤3.4), Moderate (3.4–5.4), or Poor (>5.4) [48].
- Positive Lymph Nodes: Low burden (≤3) vs. High burden (>3) [49].
- Tumor Size: Small (<20 mm) vs. Large (≥20 mm) [50].

Kaplan–Meier estimation and Cox proportional hazards analyses were conducted using the full cohort for each dataset to preserve complete observed survival information and ensure clinically interpretable group-wise comparisons. Cox models were reported with hazard ratios (HRs), corresponding 95% confidence intervals (CIs), and Wald test *p*-values to quantify effect size and statistical significance. Kaplan–Meier curves were presented with 95% confidence bands, median survival times with 95% CIs (when estimable), and log-rank *p*-values to provide a comprehensive summary of survival differences between groups [51]. The use of the full cohort for survival estimation did not introduce data leakage into the predictive modeling process. No data balancing, reweighting, or synthetic resampling was performed, allowing group distributions to reflect real-world clinical prevalence.

**Figure 1:**
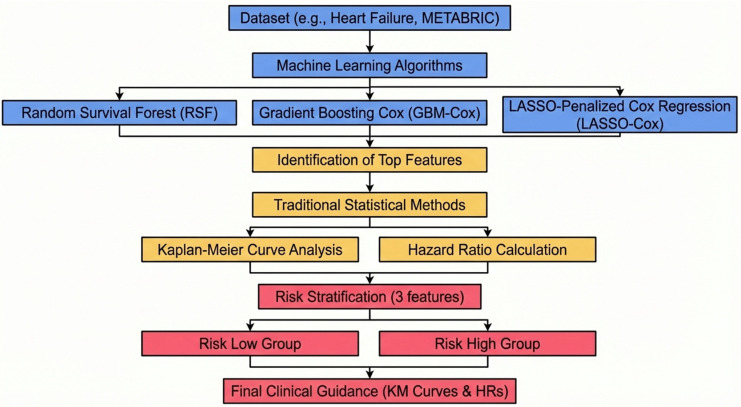
Unified Workflow: From ML Feature Selection to Clinically Interpretable Risk Stratification via Cox HR and Kaplan–Meier Curves. Note. The process progresses from machine learning–driven feature selection in high-dimensional data (blue), to identification and statistical validation of key prognostic variables (yellow) and culminates in clinical risk group classification (red).

Finally, composite risk groups (low vs. high risk) were constructed by combining three clinically significant features identified through the machine learning and validation process (Figure 1). The selection of three predictors was intentional, balancing robustness with clinical practicality. This approach enables meaningful separation of survival risk while maintaining group sizes as adequate as possible, thereby supporting model stability and real-world applicability.

Survival differences between risk groups were evaluated using hazard ratios, Kaplan–Meier curves, and log-rank testing. The Heart Failure dataset served as the primary analysis, with the METABRIC cohort used for external exploratory validation. Together, these analyses demonstrate concordance between machine learning-derived feature importance and clinically interpretable survival effects.

## 3. Result

### 3.1. Heart Failure Analysis

**Figure 2:**
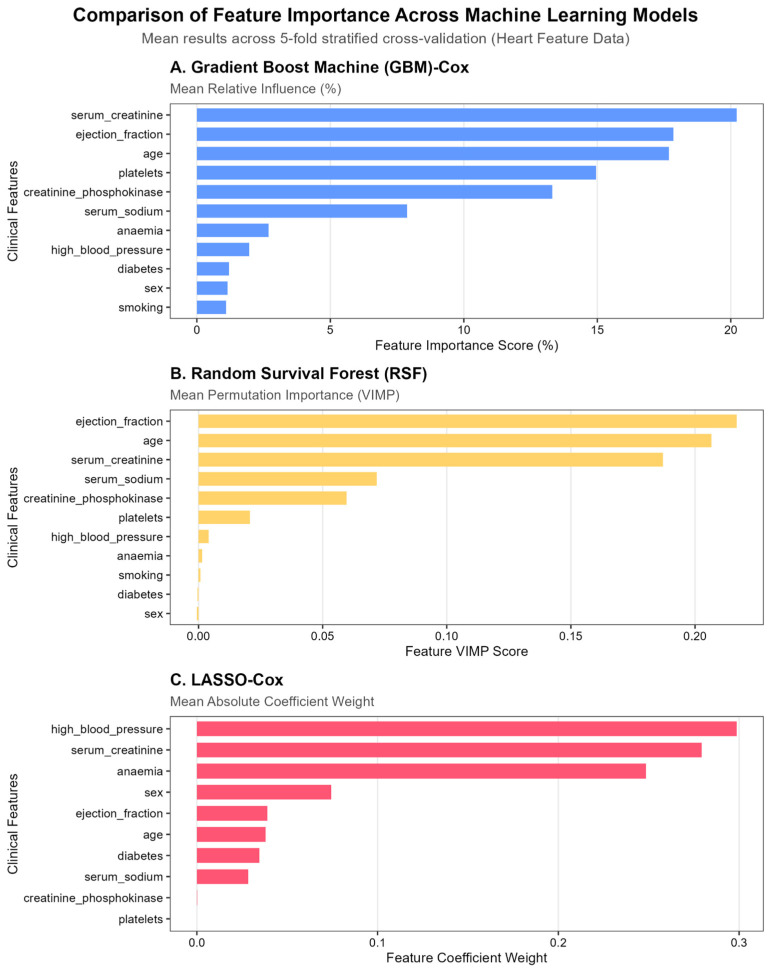
Heart Failure Data Set: Cross-Model Feature Importance Comparison (GBM Relative Influence, RSF VIMP, LASSO Coefficients). Note. Bar charts display the top 10 predictive features identified by (**A**) Gradient Boosting Machine–Cox (GBM-Cox), (**B**) Random Survival Forest (RSF), and (**C**) LASSO-Cox. Bars represent mean importance values aggregated across 5-fold stratified cross-validation. Model-specific importance metrics include relative influence (%) for GBM, permutation importance (VIMP) for RSF, and absolute coefficient weights for LASSO. Features are ordered by descending importance within each model.

We evaluated feature importance across three survival modeling approaches: GBM-Cox, RSF, and LASSO-Cox, using a 5-fold cross-validation framework. Feature importance values represent mean results across folds rather than a single data split. Across the tree-based models (GBM and RSF), serum creatinine, ejection fraction, and age consistently emerged as the most influential predictors of overall survival. In contrast, the LASSO-Cox model identified high blood pressure, serum creatinine, and anemia as the primary predictors (Figure 2). Notably, the prominence of serum creatinine and ejection fraction as key prognostic factors aligns with prior evidence in heart failure survival research [6].

The observed variation in selected features reflects the distinct algorithmic structures of the modeling approaches. Tree-based models can capture nonlinear relationships and higher-order interactions among variables, whereas LASSO imposes coefficient shrinkage and promotes a sparse linear representation. Consequently, LASSO may retain high blood pressure as a single representative marker of cardiovascular risk while shrinking correlated predictors, such as ejection fraction, toward zero.

The Heart Failure dataset included 299 patients, of whom 96 (32.1%) experienced death during the 196-day follow-up period. To assess the stability and robustness of feature selection across modeling approaches, we examined the consistency of feature rankings across the 5-fold cross-validation framework. Full performances are provided in Table 3.

In terms of overall discrimination, the GBM-Cox model achieved the highest concordance (C-index: 0.794), followed by Random Survival Forest (RSF) (0.747) and LASSO-Cox (0.690). In contrast, when discrimination was evaluated using time-dependent AUC across the follow-up period, RSF demonstrated the strongest performance (AUC: 0.782), slightly exceeding GBM-Cox (0.763), while LASSO-Cox showed comparatively lower discrimination (0.723).

Prediction error, quantified via the Integrated Brier Score (IBS), was lowest for RSF (0.158), followed by GBM-Cox (0.178), with LASSO-Cox showing higher prediction error (0.199). Taken together, these findings suggest that although GBM-Cox achieved superior global ranking ability, RSF demonstrated stronger time-specific discrimination and improved overall calibration.

Across all models, C-index values aligned with established clinical benchmarks (approximately 0.68–0.88) [6,19], indicating that all three models achieved acceptable predictive performance. Consistent with the study objective, we prioritized stable, interpretable feature identification over maximal performance optimization. By intersecting machine learning-derived importance rankings with clinical relevance, age, serum creatinine, and high blood pressure were identified as the primary predictors for further downstream survival validation.

Age and serum creatinine were consistently ranked among the top predictors by both non-linear tree-based models (GBM-Cox and RSF), indicating stable and robust associations with survival time. High blood pressure was additionally identified as a leading predictor in the LASSO-Cox model and represents a routinely monitored clinical variable, aligning with the framework’s objective of early risk identification and practical patient guidance.

However, ejection fraction revealed a divergence between machine learning–based importance and traditional regression analysis. Although highly ranked by GBM and RSF, ejection fraction did not reach statistical significance in the univariable Cox model (HR = 1.25, *p* = 0.4066). This discrepancy suggests that its association with survival may be driven by non-linear effects or higher-order interactions not fully captured within a linear proportional hazards framework. Given the study’s emphasis on clinically interpretable and statistically stable predictors for the final risk model, ejection fraction was not included in the composite risk stratification.

**Figure 3:**
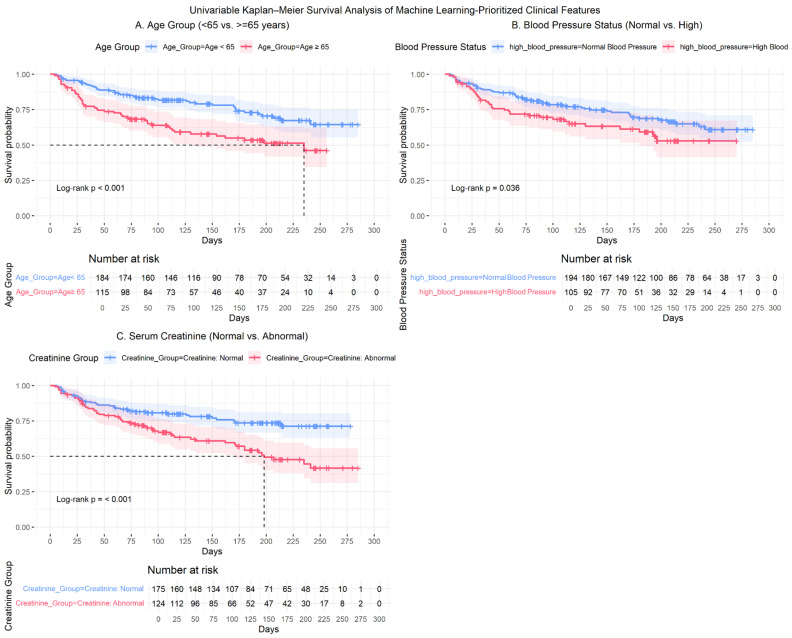
Heart Failure: Kaplan–Meier Survival Curves by Key Clinical Predictors (**A**) Age group (<65 vs. ≥65 years); (**B**) Blood pressure status (normal vs. high); (**C**) Serum creatinine level (normal vs. abnormal). Note. Shaded regions represent 95% confidence intervals. Tick marks indicate censored observations. Between-group differences were evaluated using the log-rank test, with corresponding *p*-values shown within each panel. Numbers at risk are displayed below each plot. Horizontal dashed lines indicate the 50% survival probability, and vertical dashed lines denote the corresponding median survival time.

**Figure 4:**
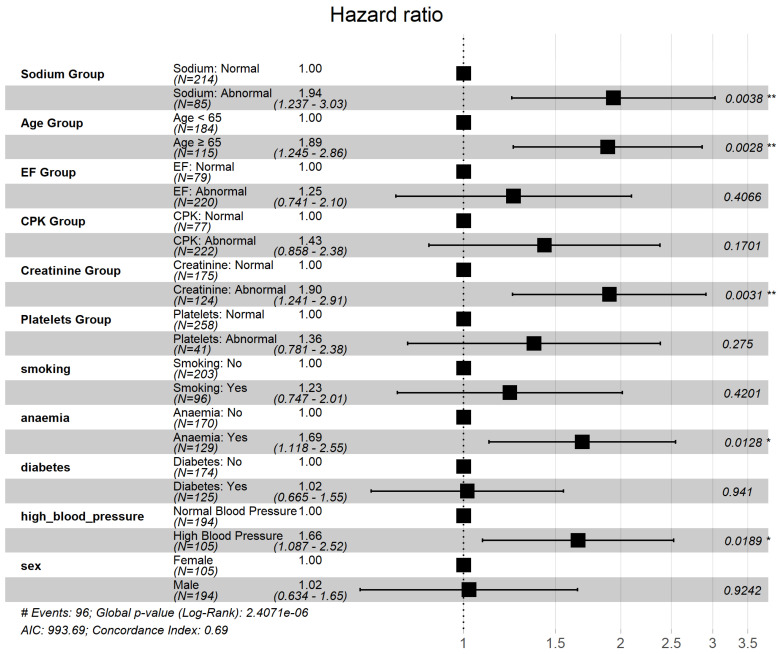
Heart Failure: Univariable Cox Hazard Ratio for ML-Prioritized Clinical Predictors (Forest Plot). Note. Squares represent hazard ratio estimates and horizontal lines indicate 95% confidence intervals; the vertical dashed line denotes a hazard ratio of 1. Two-sided *p*-values are denoted on the right. * *p* < 0.05,** *p* < 0.01.

Using the clinical categorization criteria defined in the Methods (Section 2.4), we performed univariable Cox proportional hazards analyses to validate the prognostic relevance of the machine learning–identified features. Age (≥65 vs. <65 years), serum creatinine (Abnormal vs. Normal), and high blood pressure (Yes vs. No) were each significantly associated with overall survival and satisfied with the proportional hazards assumption based on Schoenfeld residual testing. The corresponding Kaplan–Meier curves (Figure 3) showed clear and non-crossing separation between groups, further supporting the stability of the estimated effects. Hazard ratios with 95% confidence intervals are summarized in Table 4 and visualized in Figure 4.

The forest plot (Figure 4) demonstrates that older age (≥65 years) and abnormal serum creatinine were associated with the largest relative increases in mortality risk, while high blood pressure showed a more modest but statistically significant effect. These results confirm that the variables prioritized by the machine learning models retain statistical significance within a conventional survival regression framework.

**Table 4:** Heart Failure: Survival Analysis of Clinical Features (Age, Creatinine, Blood Pressure).

Feature	Category	Median Survival (95% CI)	HR (95% CI)	*p*-Value
Age	<65 (*n* = 184)	NR		
	≥65 (*n* = 115)	235.0 [130.0, NR]	1.89 [1.25, 2.86]	0.0028
Serum Creatinine	Normal (*n* = 175)	NR		
	Abnormal (*n* = 124)	198.0 [171.0, NR]	1.90 [1.24, 2.91]	0.0031
Blood Pressure	Normal (*n* = 194)	NR		
	High (*n* = 105)	NR	1.66 [1.09, 2.52]	0.0189

Note. Median survival times (in days) were estimated using the Kaplan–Meier method. Hazard ratios (HRs) and corresponding 95% confidence intervals (CIs) were derived from univariable Cox proportional hazards regression models, with the first category of each variable used as the reference group. *p*-values correspond to the Wald test from the Cox model. NR indicates that the median survival was not reached during follow-up.

Kaplan–Meier survival curves further illustrated clear separation between clinically defined subgroups (Figure 3). Patients aged ≥65 years and those with abnormal serum creatinine exhibited visibly reduced survival compared with their respective reference groups. In addition, survival differences between blood pressure subgroups also demonstrated a statistically significant separation. Median survival times, hazard ratios, and log-rank *p*-values are reported in Table 4 to provide complete numerical detail.

Collectively, these findings indicate that age, serum creatinine, and blood pressure define clinically distinct risk strata, reinforcing the prognostic relevance of the machine learning–identified features. These individual effects motivated the construction of the composite risk stratification model presented in the following section.

Based on these validated individual effects, a composite risk model was constructed. The low-risk group (*n* = 71) consisted of patients aged <65 years with normal serum creatinine and normal blood pressure; all remaining patients were classified as high risk (*n* = 228).

**Figure 5:**
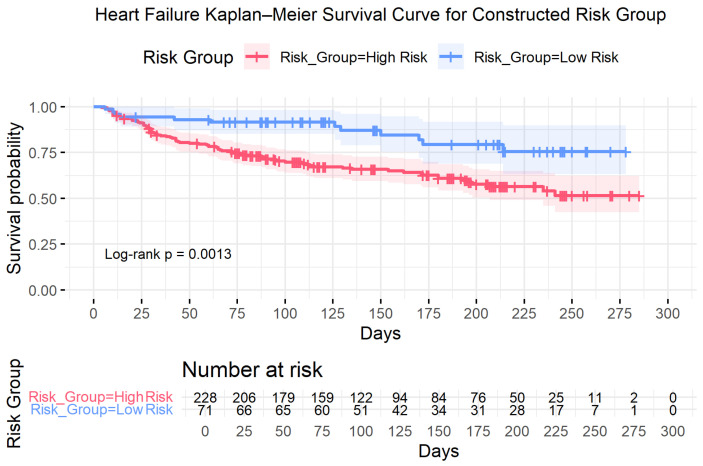
Heart Failure: Kaplan–Meier Survival Curves for Constructed Risk Group (Low vs. High Risk). Note. The low-risk group consisted of patients aged <65 years with Normal Serum Creatinine and Normal Blood Pressure; all remaining patients were classified as high risk. Shaded regions represent 95% confidence intervals. Tick marks indicate censored observations. Between-group differences were evaluated using the log-rank test, with corresponding *p*-values shown within the panel. Numbers at risk are displayed below the plot.

Kaplan–Meier analysis demonstrated clear separation between the survival trajectories of the two groups (Figure 5). In univariable Cox regression, the high-risk group exhibited a hazard ratio of 2.61 (*p* = 0.0013) compared with the low-risk group, corresponding to more than a twofold increase in mortality risk. Median survival was not reached in either group during follow-up, consistent with the persistent separation of survival probabilities over time. Detailed survival estimates are provided in Table 5.

Together, these findings illustrates that integrating machine learning-based feature selection with conventional survival analysis can produce a transparent and clinically interpretable risk stratification framework. Using three routinely available variables, age, serum creatinine, and blood pressure, the framework enables early identification of heart failure patients at elevated risk, thereby supporting targeted monitoring and management while maintaining interpretability for clinical decision-making.

### 3.2. METABRIC Breast Cancer Analysis

To evaluate the generalizability of the proposed framework, the identical feature selection and survival analysis pipeline was applied to the METABRIC breast cancer cohort as an independent validation dataset. Feature importance rankings were averaged across the 5-fold cross-validation framework to assess selection stability and reduce the influence of partition-specific variability.

**Figure 6:**
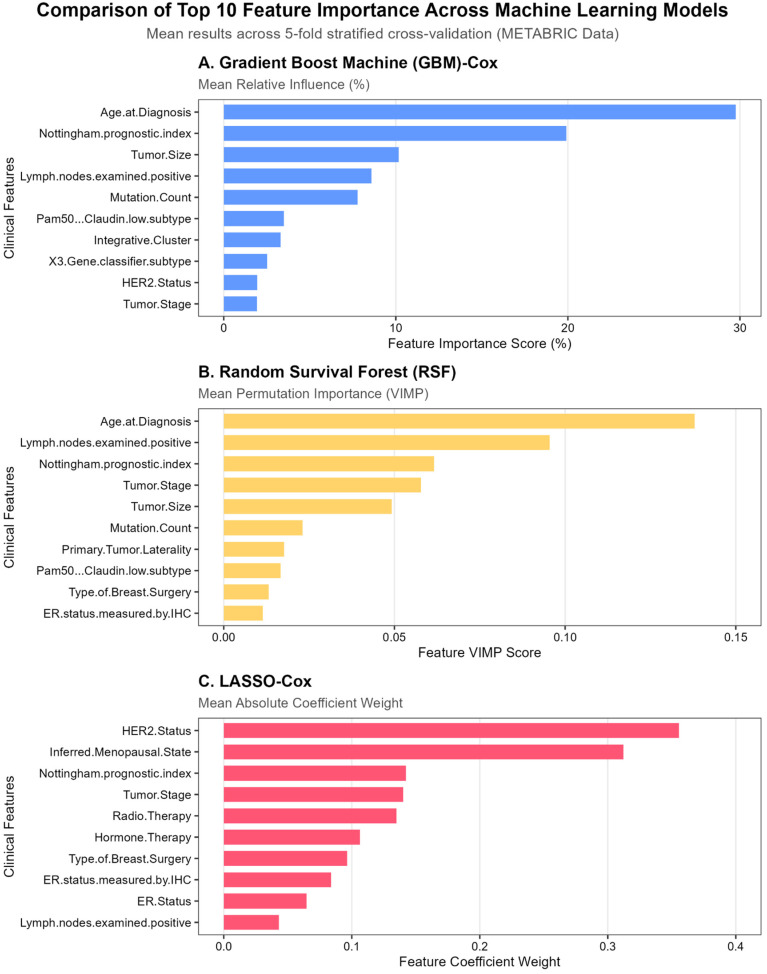
METABRIC: Cross-Model Feature Importance Comparison (GBM, RSF, LASSO) Highlighting Shared and Model-Specific Predictors. Note. Bar charts display the top 10 predictive features identified by (**A**) Gradient Boosting Machine–Cox (GBM-Cox), (**B**) Random Survival Forest (RSF), and (**C**) LASSO-Cox. Bars represent mean importance values aggregated across 5-fold stratified cross-validation. Model-specific importance metrics include relative influence (%) for GBM, permutation importance (VIMP) for RSF, and absolute coefficient weights for LASSO. Features are ordered by descending importance within each model.

Consistent with the Heart Failure analysis, partial agreement in feature importance was observed across models (Figure 6). The non-linear tree-based models (GBM-Cox and RSF) showed strong concordance, consistently ranking age at diagnosis, Nottingham Prognostic Index (NPI), and number of positive lymph nodes among the most influential predictors of overall survival.

In contrast, the LASSO-Cox model emphasized a different subset of variables, prioritizing HER2 status, inferred menopausal state, and Radio Therapy. Nevertheless, NPI emerged as a relatively high-ranked feature in LASSO-Cox models, indicating a shared prognostic signal across modeling paradigms despite differences in feature selection strategies. Moreover, several top-ranked features identified across in tree-based and linear models, including NPI and HER2 status, are well-established and clinically validated prognostic factors in breast cancer [32,34,52].

The observed variation in feature rankings across models likely reflects both algorithmic differences and the increased dimensionality of the METABRIC dataset, which includes a larger number of correlated tumor- and treatment-related variables. Nevertheless, despite differences in ranking order, the models consistently identified overlapping core predictors. This convergence on a shared subset of clinically meaningful features supports the robustness and generalizability of the proposed framework.

The METABRIC cohort included 1310 patients with complete data, of whom 743 (56.7%) experienced death during a maximum follow-up of 396 months. Full performance estimates are provided in Table 6.

Overall discrimination, assessed using Harrell’s C-index across the full follow-up period, was highest for GBM-Cox (0.711), followed by RSF (0.687) and LASSO-Cox (0.665). To ensure a clinically relevant and statistically stable assessment, time-dependent AUC and Integrated Brier Score (IBS) were evaluated at a 5-year (60-month) horizon. This time point was selected to align with standard oncological reporting conventions and to mitigate numerical instability associated with heavy censoring at later follow-up intervals [44,53].

At the 5-year assessment, RSF demonstrated the strongest discrimination (AUC: 0.752) and the lowest prediction error (IBS: 0.151), followed by GBM-Cox (AUC: 0.734) and LASSO-Cox (AUC: 0.700). This pattern mirrors the findings in the Heart Failure dataset, where GBM-Cox achieved the highest overall C-index, whereas RSF exhibited comparatively stronger time-specific discrimination and calibration. Together, these results indicate consistent performance characteristics across disease contexts: GBM-Cox demonstrates strong global ranking ability, while RSF provides stable time-dependent performance.

Overall, the observed discrimination levels are consistent with prior breast cancer survival studies, where C-index values typically within the range (0.6–0.8) [31,32,54], indicating that model performance meets established clinical benchmarks. The slightly lower discrimination compared with the Heart Failure dataset is expected given the greater dimensionality, tumor heterogeneity, and treatment complexity of the METABRIC cohort.

Having demonstrated adequate performance across two distinct clinical domains, the emphasis of the framework shifts from maximizing predictive accuracy to connecting machine learning-based feature selection with conventional survival analysis. This unified strategy enables statistically sound and clinically interpretable risk stratification, which represents the central contribution of the present study.

**Figure 7:**
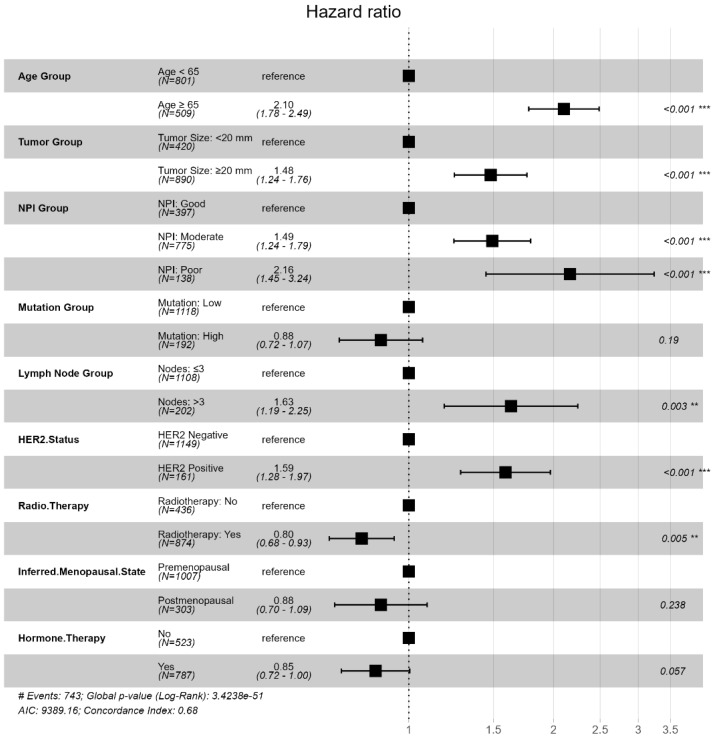
METABRIC: Multivariate Cox Proportional Hazards Ratios for Selected Prognostic Predictors. Note. Squares represent hazard ratio estimates and horizontal lines indicate 95% confidence intervals; the vertical dashed line denotes a hazard ratio of 1. Two-sided *p*-values are denoted on the right. ** *p* < 0.01, *** *p* < 0.001.

We next evaluated the clinical significance of the machine learning-identified predictors using traditional survival modeling approaches. Based on the predefined clinical categorization criteria (Section 2.4), univariable Cox proportional hazards analyses were conducted to validate the prognostic relevance (Figure 7; Table 7). Inferred menopausal status was excluded due to lack of statistical significance (*p* = 0.238). Tumor size and lymph node status were not modeled separately to avoid multicollinearity, as both are intrinsic components of the Nottingham Prognostic Index (NPI) [48], defined as NPI = (0.2 × tumor size [cm]) + lymph node score + histological grade. Therefore, NPI was retained as a composite prognostic measure to preserve parsimony while capturing tumor burden information.

**Figure 8:**
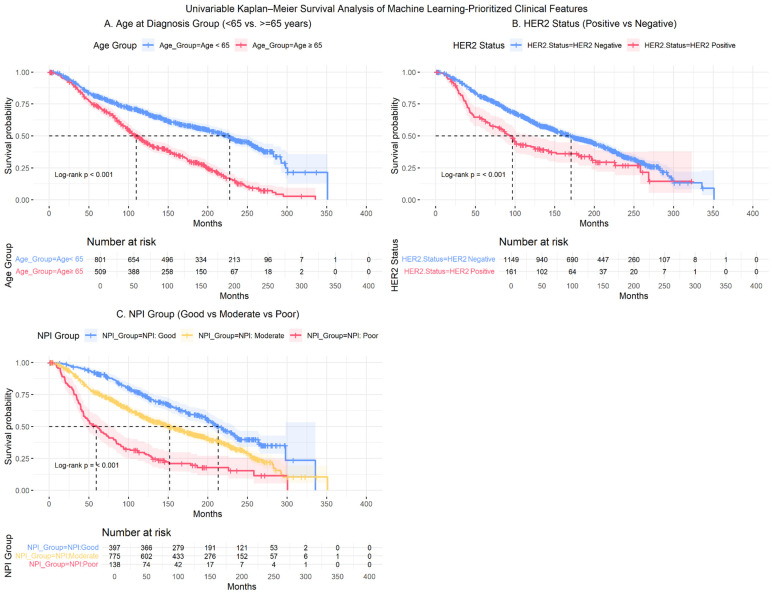
METABRIC: Kaplan–Meier Survival Curves by Final Predictors (**A**) Age at diagnosis (<65 vs. ≥65 years); (**B**) HER2 Status (Negative vs. Positive); (**C**) NPI Group (Good vs. Moderate vs. Poor). Note. Shaded regions represent 95% confidence intervals. Tick marks indicate censored observations. Between-group differences were evaluated using the log-rank test, with corresponding *p*-values shown within the panel. Numbers at risk are displayed below the plot. Horizontal dashed lines indicate the 50% survival probability, and vertical dashed lines denote the corresponding median survival time.

To construct downstream risk groups, the three most influential and clinically validated predictors, age at diagnosis group, NPI group, and HER2 status, were retained. Univariable Cox analysis confirmed that all three variables were significantly associated with overall survival (Table 7; Figure 7). Increasing age, worsening NPI category, and HER2-positive status were each associated with elevated mortality risk. Furthermore, all three variables satisfied the proportional hazards assumption based on Schoenfeld residual testing. The corresponding Kaplan–Meier curves (Figure 8) showed clear and non-crossing separation between groups, further supporting the stability of the estimated effects.

Patients aged at diagnosis ≥65 years had more than twice the mortality risk compared with younger patients (HR = 2.10), and those in the Poor NPI category demonstrated markedly increased hazard relative to the Good group (HR = 2.16). HER2-positive status was also associated with worse survival (HR = 1.59).

Kaplan–Meier analyses further demonstrated clear and statistically significant separation across all three variables in the METABRIC cohort (Figure 8; log-rank *p* < 0.001 for all selected features) which is consistent with the findings in Heart Failure Data. Median survival was substantially shorter among patients age at diagnosis ≥65 years compared with those <65 years (109.8 vs. 227.7 months) (Figure 3A) and among HER2-positive patients compared with HER2-negative patients (96.6 vs. 170.7 months) (Figure 3B). A graded survival pattern was observed across NPI categories, with median survival decreasing from 213.1 months (Good) to 152.1 months (Moderate) and 59.5 months (Poor) (Figure 3C). Detailed subgroup sample size, hazard ratios with associated *p*-values, and median survival times with corresponding 95% confidence intervals are summarized in Table 7.

**Figure 9:**
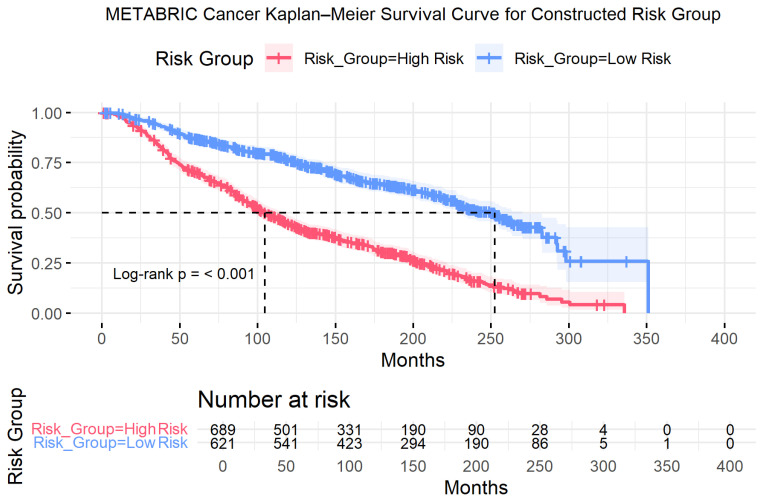
METABRIC: Kaplan–Meier Survival Curves for Constructed Risk Group (Low vs. High Risk). Note. The low-risk group consisted of patients aged <65 years at diagnosis with Good or Moderate NPI and HER2-negative status; all remaining patients were classified as high risk. Shaded regions represent 95% confidence intervals. Tick marks indicate censored observations. Between-group differences were evaluated using the log-rank test, with corresponding *p*-values shown within each panel. Numbers at risk are displayed below each plot. Horizontal dashed lines indicate the 50% survival probability, and vertical dashed lines denote the corresponding median survival time.

Finally, A composite risk stratification rule was constructed using the three validated prognostic features (Figure 9). Patients were classified as Low Risk (*n* = 621) if they were aged <65 years at diagnosis, had Good or Moderate NPI, and were HER2-negative. All remaining patients presenting with at least one unfavorable factor were categorized as High Risk (*n* = 689).

Kaplan–Meier analysis demonstrated clear and statistically significant separation between the two groups (log-rank *p* < 0.001) (Figure 9), confirming the robustness of the composite stratification. Patients in the High-Risk group experienced substantially poorer survival compared with those in the Low-Risk group (HR = 2.73). Median survival differed markedly between groups: 104.7 months in the High-Risk group versus 252.3 months in the Low-Risk group, corresponding to an absolute survival difference of 147.6 months (approximately 12.3 years). Moreover, this survival difference reflects prognostic risk stratification based on baseline clinical features rather than any treatment effect. Detailed survival estimates, hazard ratios, confidence intervals, and subgroup sample sizes are summarized in Table 8.

In summary, the METABRIC analysis validates the proposed framework within a large and complex dataset. By integrating machine-learning-derived feature importance with established clinical prognostic factors, the approach translates data-driven signals into an interpretable and clinically actionable risk stratification model. Importantly, feature importance is not treated as an abstract ranking metric; rather, it is converted into quantifiable survival differences, expressed through hazard ratios and absolute median survival gains. This unified framework achieves clear separation of survival outcomes and supports individualized prognostic counseling, enabling clinicians to tailor follow-up and surveillance strategies based on age at diagnosis, NPI category, and HER2 status.

## 4. Discussion

This study demonstrates the value of integrating machine-learning-based feature selection with conventional survival analysis to achieve clinically interpretable risk stratification. Using heart failure as the primary application and breast cancer as external validation, the proposed framework systematically identifies the most influential prognostic features and translates them into quantifiable survival benefits, expressed through hazard ratios and absolute differences in median survival time.

Across both diseases, three dominant predictors enabled clear separation of low- and high-risk groups, with substantial differences in survival time observed between strata. In the heart failure cohort, age, serum creatinine, and blood pressure were incorporated into a simplified rule based on routinely collected clinical measurements, demonstrating that meaningful prognostic discrimination can be achieved without reliance on opaque or highly complex models. In the METABRIC cohort, age at diagnosis, Nottingham Prognostic Index, and HER2 status similarly produced clear survival stratification in a larger and more heterogeneous dataset, reinforcing the adaptability of the approach.

The main contributions of this study are threefold. First, we established a systematic framework that integrates cross-validated machine-learning feature selection with conventional survival modeling, promoting transparency and methodological rigor in clinical prediction. Second, we convert machine-learning-derived feature importance into quantifiable survival time benefits, expressed as hazard ratios and absolute median survival differences, thereby bridging predictive modeling and practical decision-making. Third, we empirically validate the robustness and generalizability of our model structure across two clinically distinct datasets, demonstrating the framework potential disease-agnostic applicability for survival risk profiling.

Several limitations should be acknowledged. First, the heart failure dataset [36] used in this study contains a relatively small number of observations and a limited set of predictors. Validation using additional heart failure cohorts with larger sample sizes and richer clinical information would further strengthen the robustness of these findings. Nevertheless, validation in the METABRIC dataset, which features substantially greater dimensionality and complexity, provides evidence for the broader applicability of the proposed framework.

Second, although machine learning methods were employed for feature selection, several influential predictors were continuous variables that required categorization to enhance clinical interpretability. While this step may result in some information loss, all cut points were defined a priori based on established clinical guidelines [6] to minimize bias. This highlights the continued importance of clinical expertise in translating data-driven findings into practically useful and actionable tools.

Finally, a slight difference in predictive discrimination was observed between datasets, with stronger performance in the heart failure cohort than in the METABRIC cohort. This variation likely reflects the greater biological heterogeneity of breast cancer and the complexity of long-term oncologic survival outcomes. Future work may extend the proposed framework to incorporate high-dimensional genomic and molecular data within the METABRIC dataset and similar cohorts. Integrating omics-level predictors with clinical variables, while preserving interpretability and reproducibility, may further enhance prognostic precision. Such extensions could involve structured or hybrid modeling strategies designed to balance high-dimensional feature selection with transparent survival estimation.

Despite these limitations, the proposed framework effectively bridges machine learning-based feature discovery with traditional survival analysis. By combining non-linear models, such as Random Survival Forests and GBM-Cox, with penalized regression and validating results using standard Cox regression and Kaplan–Meier analysis, the framework balances diagnostic performance with transparency and clinical relevance.

## 5. Conclusions

This study presents an interpretable and reproducible framework that connects machine-learning-based feature selection with conventional survival analysis to support prognostic risk stratification. Across both heart failure and METABRIC breast cancer cohorts, the approach consistently translated machine-learning-derived importance measures into clinically meaningful biomarkers and quantifiable survival time benefits, expressed through hazard ratios and absolute differences in median survival time.

By prioritizing interpretability and clinical applicability rather than predictive performance alone, the proposed methodology provides a practical pathway for integrating machine learning into patient risk profiling and longitudinal monitoring. Importantly, the framework moves beyond ranking variables to demonstrating measurable survival gains between risk groups, enabling clearer prognostic communication and more informed clinical decision-making. Together, these findings reinforce the value of transparent, clinically grounded analytics in advancing precision diagnostics and patient-centered care.

## Figures and Tables

**Table 1:** Heart Failure Cohort: Clinical Feature (N = 299; 11 Variables).

Variable Type	Heart Failure Data Variables
Continuous (6)	Age, Creatinine Phosphokinase, Ejection Fraction, Platelets, Serum Creatinine, Serum Sodium
Binary (5)	Anaemia, Diabetes, High blood pressure, Sex, Smoking

**Table 2:** METABRIC Cohort: Clinical and Genomic Feature (N = 1310; 20 Variables).

Variable Type	METABRIC Variables
Continuous (4)	Age at Diagnosis, Lymph nodes examined positive, Mutation Count, Tumor Size
Binary (9)	ER Status, PR status, HER2 Status, Chemotherapy, Hormone Therapy, Radio Therapy, Type of Breast Surgery, Inferred Menopausal State, Primary Tumor Laterality
Categorical (7)	Cancer Type Detailed, Cellularity, Pam50+ Claudin-low subtype, Tumor Other Histologic Subtype, 3-Gene classifier subtype, Neoplasm Histologic Grade, Tumor Stage

**Table 3:** Heart Failure: Cross-Validated Predictive Performance of GBM-Fox, RSF, and LASSO-Cox (C-index, AUC, IBS).

	Harrell’s C-Index (95% CI)	Integrated Brier Score (IBS) (95% CI)	AUC (95% CI)
GBM-Cox	0.794 [0.756, 0.832]	0.178 [0.161, 0.196]	0.763 [0.695, 0.831]
RSF	0.747 [0.727, 0.766]	0.158 [0.144, 0.172]	0.782 [0.718, 0.846]
LASSO-Cox	0.690 [0.643, 0.738]	0.199 [0.154, 0.244]	0.723 [0.622, 0.823]

Note. Model discrimination and prediction accuracy were evaluated using Harrell’s concordance index (C-index), time-dependent area under the curve (AUC), and Integrated Brier Score (IBS). Higher C-index and AUC indicate better discrimination, whereas lower IBS reflects improved overall prediction accuracy. IBS was calculated over the interval corresponding to the 75th of observed event times. Estimates represent mean performance across five-fold stratified cross-validation, with 95% confidence intervals obtained via fold-wise variability using t-distribution.

**Table 5:** Heart Failure: Survival Contrast for Composite Risk Rule (Low vs. High Risk) Using Cox HR and Kaplan–Meier Medians.

Risk Group	Median Survival (95% CI)	HR (95% CI)	*p*-Value
Low Risk (*n* = 71)	NR		
High Risk (*n* = 228)	NR	2.61 [1.42–4.78]	0.0013

Note. Median survival (days) was estimated using the Kaplan–Meier method. Hazard ratios (HRs) and corresponding 95% confidence intervals (CIs) were derived from an univariable Cox proportional hazards model, with the low-risk group serving as the reference category. The *p*-value corresponds to the Wald test from the Cox model. NR indicates that the median survival was not reached during follow-up.

**Table 6:** METABRIC: Cross-Validated Predictive Performance of GBM-Cox, RSF, and LASSO-Cox.

	Harrell’s C-Index (95% CI)	Integrated Brier Score (IBS) (95% CI)	AUC (95% CI)
GBM-Cox	0.711 [0.698, 0.724]	0.177 [0.146, 0.209]	0.734 [0.673, 0.795]
RSF	0.687 [0.670, 0.703]	0.151 [0.137, 0.164]	0.752 [0.681, 0.823]
LASSO-Cox	0.665 [0.653, 0.678]	0.153 [0.140, 0.166]	0.700 [0.659, 0.742]

Note. Overall model discrimination is summarized using Harrell’s concordance index (C-index), while 5-year predictive performance is assessed using the Integrated Brier Score (IBS) and 5-year time-dependent area under the curve (AUC). Estimates represent mean performance across five-fold stratified cross-validation, with 95% confidence intervals obtained via fold-wise variability using t-distribution.

**Table 7:** METABRIC: Survival Analysis of Clinical Features (Age, NPI, HER2).

Feature	Category	Median Survival (95% CI)	HR (95% CI)	*p*-Value
Age	<65 (*n* = 801)	227.7 [204.2, 249.5]		
	≥65 (*n* = 509)	109.8 [101.1, 119.5]	2.10 [1.25, 2.86]	<0.001
NPI Group	Good (*n* = 397)	213.1 [199.4, 233.9]		
	Moderate (*n* = 775)	152.1 [134.5, 170.7]	1.49 [1.24, 1.79]	<0.001
	Poor (*n* = 178)	59.5 [44.8, 80.7]	2.16 [1.45, 3.24]	<0.001
HER2 Status	Negative (*n* = 1149)	170.7 [155.7, 187.8]		
	Positive (*n* = 161)	96.6 [75.4, 124.1]	1.59 [1.28, 1.97]	<0.001

Note. Median survival times (in Months) were estimated using the Kaplan–Meier method. Hazard ratios (HRs) and corresponding 95% confidence intervals (CIs) were derived from univariable Cox proportional hazards regression models, with the first category of each variable used as the reference group. *p*-values correspond to the Wald test from the Cox model.

**Table 8:** METABRIC: Survival Contrast for Composite Risk Rule (Low vs. High Risk) Using Cox HR and Kaplan–Meier Medians.

Risk Group	Median Survival (95% CI)	HR (95% CI)	*p*-Value
Low Risk (*n* = 621)	104.7 [97.8, 116.2]		
High Risk (*n* = 689)	252.3 [227.9, 270.1]	2.73 [2.34–3.19]	<0.001

Note. Median survival (months) was estimated using the Kaplan–Meier method. Hazard ratios (HRs) and corresponding 95% confidence intervals (CIs) were derived from the univariable Cox proportional hazards model, with the low-risk group serving as the reference category. The *p*-value corresponds to the Wald test from the Cox model.

## Data Availability

The original data presented in the study are openly available in METABRIC breast cancer dataset at [https://www.cbioportal.org/study/clinicalData?id=brca_metabric, (accessed on 1 January 2026)] and Heart failure dataset at [36].
